# Differentiation of two swim bladdered fish species using next generation wideband hydroacoustics

**DOI:** 10.1038/s41598-021-89941-7

**Published:** 2021-05-18

**Authors:** Sarah M. Gugele, Marcus Widmer, Jan Baer, J. Tyrell DeWeber, Helge Balk, Alexander Brinker

**Affiliations:** 1 Fisheries Research Station Baden-Württemberg (LAZBW), Argenweg 50/1, 88085 Langenargen, Germany; 2 Department of Physics, University of Oslo, Blindern, PO. Box. 1048, 0317 Oslo, Norway; 3 Institute for Limnology, University of Constance, Mainaustraße 252, 78464 Konstanz, Germany

**Keywords:** Freshwater ecology, Invasive species

## Abstract

Monitoring fish populations in large, deep water bodies by conventional capture methodologies requires intensive fishing effort and often causes mass mortality of fish. Thus, it can be difficult to collect sufficient data using capture methods for understanding fine scale community dynamics associated with issues such as climate change or species invasion. Hydroacoustic monitoring is an alternative, less invasive technology that can collect higher resolution data over large temporal and spatial scales. Monitoring multiple species with hydroacoustics, however, usually requires conventional sampling to provide species level information. The ability to identify the species identity of similar-sized individuals using only hydroacoustic data would greatly expand monitoring capabilities and further reduce the need for conventional sampling. In this study, wideband hydroacoustic technology was used in a mesocosm experiment to differentiate between free swimming, similar-sized individuals of two swim-bladdered species: whitefish (*Coregonus wartmanni*) and stickleback (*Gasterosteus aculeatus*). Individual targets were identified in echograms and variation in wideband acoustic responses among individuals, across different orientations, and between species was quantified and visually examined. Random forest classification was then used to classify individual targets of known species identity, and had an accuracy of 73.4% for the testing dataset. The results show that species can be identified with reasonable accuracy using wideband hydroacoustics. It is expected that further mesocosm and field studies will help determine capabilities and limitations for classifying additional species and monitoring fish communities. Hydroacoustic species differentiation may offer novel possibilities for fisheries managers and scientists, marking the next crucial step in non-invasive fish monitoring.

## Introduction

Fish communities are subject to a variety of influences and stressors, and the resulting population and stock dynamics may have far reaching implications for both ecosystems and fisheries^1^. The ability of researchers and fishery managers to track and understand these changes is however limited by the difficulty in achieving reliable assessments of fish populations with a meaningful resolution in time and space^2^. Monitoring fish populations using invasive capture methods is problematic, especially in large, deep waters due to high time, labour, and material costs, and the mortality of fishes^3–6^. For example, it is very difficult to collect sufficient data to properly monitor the impacts of invasive aquatic species^7–10^ and to identify the mechanisms driving these changes using conventional sampling^3,11^. Hydroacoustic surveying is a widely used, less invasive approach that can provide highly resolved data for following trends in fish abundance, biomass, and movement that are often associated with ecosystem changes^12–16^. However, monitoring multiple species with hydroacoustics usually requires conventional sampling to provide species level information^17–19^, unless acoustic responses can be classified to species based on size differences or school morphological differences^17,20,21^. The ability to classify individual targets or aggregations of multiple species using only hydroacoustics would greatly expand monitoring capabilities and reduce the need for conventional sampling.

Hydroacoustic fish surveys use active sonar to locate fish (and other organisms) in the water column based on the principle that sound travels through water and produces backscatter when fish or other objects are encountered. Hydroacoustic surveys are most often conducted using vertical sampling where soundwaves travel downward from a boat at the surface and encounter fish dorsally. Horizontal and upward facing hydroacoustic surveys are also possible^14^. The acoustic backscatter produced is then converted to estimates of fish size, density, and population biomass^19,22,23^. Trawling^4,18,24^ or gillnetting^25–27^ is conventionally used to provide species composition data when multiple species are present. While this approach provides reliable information, conventional sampling is invasive, causes death of sampled fish, and has a lower spatial and temporal resolution than hydroacoustics. There is thus substantial interest in identifying fish species using hydroacoustic data alone, but this has only been possible under certain circumstances.

Most hydroacoustic species classification studies have used descriptors of school morphology and environmental characteristics to classify schooling pelagic species of high commercial importance^14,17,20,21,28–30^. Hydrocacoustic classification of non-schooling or partial-schooling species (such as those which school by day and disperse at night), will most likely rely on acoustic information obtained from individual fish echoes during night time surveys when fish disaggregate^4,31,32^. Species classification of individual fish targets typically relies upon vertical hydroacoustics using target strength (TS; echo amplitude created by the fish expressed as the logarithmic transformation of the backscattering cross section given in decibels) cutoffs to separate species with known size differences^14,19,32–34^. Species with and without swim-bladders can also be readily differentiated, since air in swim-bladders produces a significant acoustic response^17,35^. Imaging sonar has also been used for individual targets^36^, but for technical reasons is not as widely used in pelagic surveys as in narrower systems such as riverine corridors^37,38^ or trawl mouths^39^. It is common for similar-sized individuals of multiple species with swim bladders to be present during a survey, but hydroacoustic classification of individual targets has not yet been achieved in this case.

It is hypothesized that such classification via machine learning may be possible if morphological differences among species (e.g. swim bladder or body shape) result in sufficiently distinct acoustic responses when measured across a broad range of frequencies^40–42^. This hypothesis can now be more readily tested since recently developed, commercial wideband echosounders emit multiple frequencies across a wide frequency range of 40–50% around their nominal frequency, known as a chirp function, instead of only one or a few frequencies^43–45^. Wideband echosounders using pulse compression can further provide better signal to noise ratio and range resolution relative to narrowband echosounders^45^. Pulse compression is achieved by correlating the returned echo signal with a model of the emitted pulse^45,46^. However, single echo detection is more complicated and less reliable, and the matched filtering process introduces artificial side-lobe phenomena that may cause incorrect interpretation of the acoustic data, especially where small and large targets near boundaries, for example a lake bottom, are present. The side-lobes produced by wideband is a side effect caused by the pulse compression algorithm, and they occur as weaker peaks or shadows on each side of the main peak and can be falsely interpreted as smaller fish next to a big fish. Slow ramping may be applied to reduce side lobe effects but this also reduces the bandwidth for spectral characterization^46,47^. These potential limitations can however be overcome through careful processing, enabling a wideband echosounder to produce acoustic response curves across a wide frequency band that may provide information for species identification^45,46^.

This study explores the possibility of using wideband hydroacoustic responses to differentiate similar-sized individuals of two species that inhabit the pelagic zone of Lake Constance, Europe: native whitefish (*Coregonus* sp.) and invasive three-spined stickleback (*Gasterosteus aculeatus*, hereafter referred to as sticklebacks). The recent invasion of sticklebacks into the pelagic zone of Lake Constance^32^ has had significant negative effects on the endemic fish community and the whitefish fishery^48^. Sticklebacks display a very considerable food niche overlap with native whitefish (*Coregonus* sp.), feed on whitefish eggs and larvae^49^, and have been linked to whitefish declines in recent years^50^. High resolution spatial and temporal data for both species are urgently needed to better understand and manage invasion impacts, and hydroacoustic monitoring are strongly preferred given the aforementioned limitations of conventional sampling in large, deep water bodies like Lake Constance. It is currently possible to use TS cutoffs to differentiate much larger adult whitefish from stickleback^32^, but similar sized whitefish juveniles cannot be separated from stickleback using existing tools. Differences between the two species, including swim bladder shape and body covering (scales for whitefish and bony plates for sticklebacks), could result in sufficiently different wideband hydroacoustic responses to allow species identification of similar sized individuals. To test this hypothesis, wideband hydroacoustic data were collected using a mesocosm experiment and the random forest machine-learning method was used to determine if individual targets could be classified to species.

## Material and methods

### Experimental design

**Figure 1:**
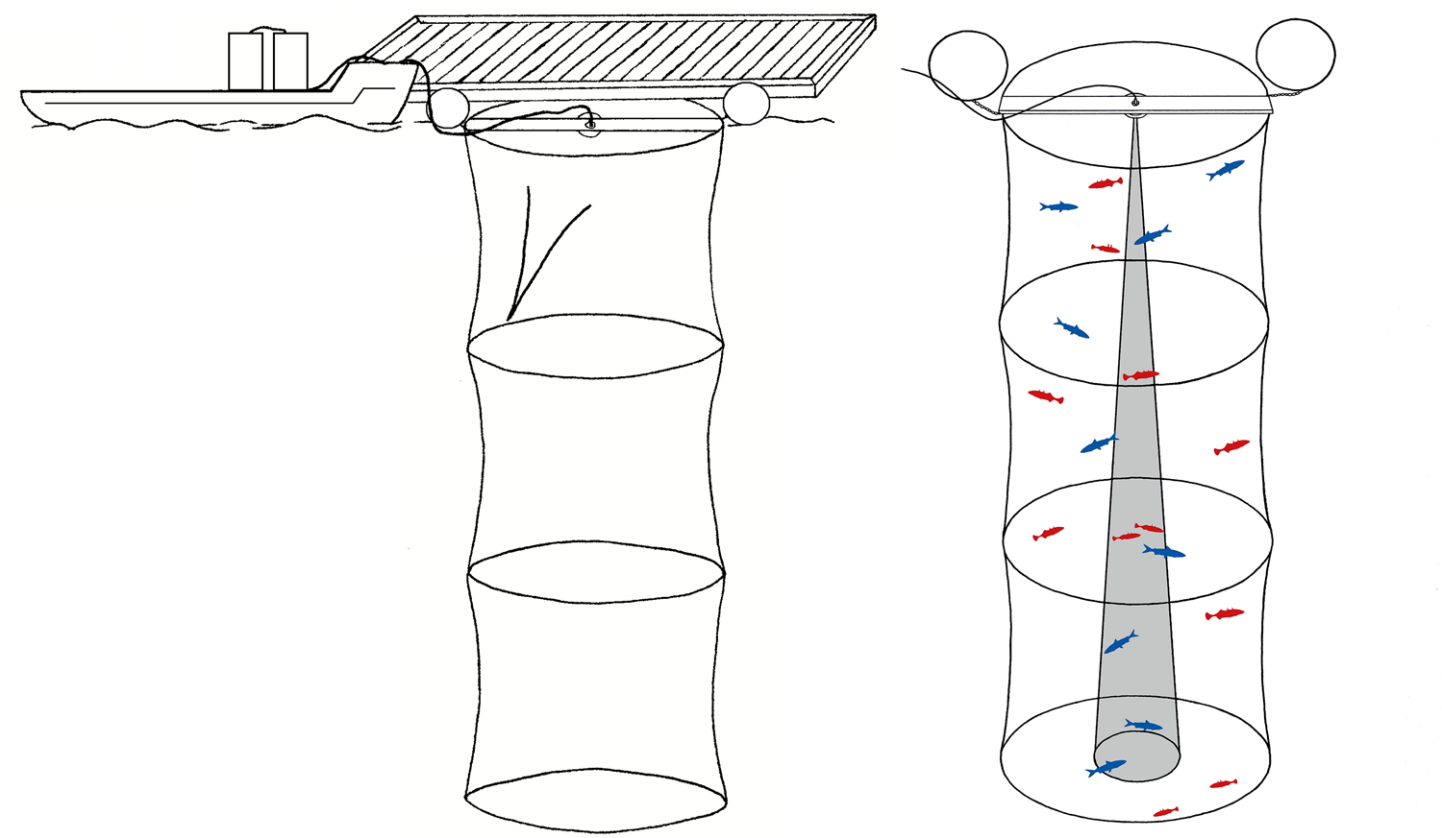
Left: experimental setup including boat with two tanks where the EK 80 wide band transceiver and a laptop were stored during the experiment and the mesocosm (6 × 2.3 m) attached to a floating pontoon. Right: mesocosm stocked with free swimming whitefish (blue) and sticklebacks (red), which are depicted together but were sampled separately for data collection. The cone (in grey) represents the true to scale sound cone emitted by the ES-120-7C transducer with an opening angle of 7°. Note that fish are not true to scale due to their small size. Drawings by Leonie Kneipp, LKdraw.

Data were collected in October 2018 using a mesocosm placed in a sheltered part of a marina of upper Lake Constance. The mesocosm comprised a 6 m high, 2.3 m diameter cylindrical net cage with a volume of around 25 m³ and a mesh size of 6 mm (Fig. 1). The cage was mounted with the cylinder length oriented vertically, suspended from buoys that ensured the top remained 10 cm below the water surface, attached by a rope to a pontoon to prevent drifting. Ropes attached to the bottom of the mesocosm were used to raise it and a zipper was used to remove and introduce fish (Fig. 1). The transducer was centered just inside the mesocosm, at the top, with the acoustic axis pointing down, emitting sound pulses from a depth of 20 cm. The EK80 wide band transceiver (Simrad, Horten, Norway) echo sounder, laptop, and power supply were located under cover on a boat moored next to the mesocosm (Fig. 1).

### Experimental animals

**Figure 2:**
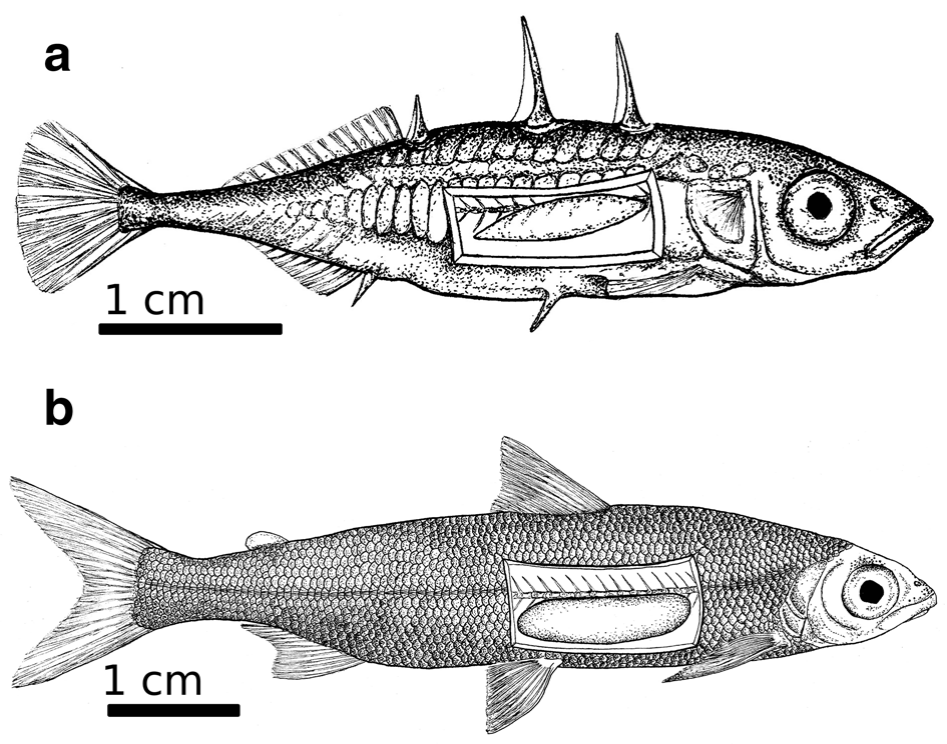
Juvenile stickleback (**a**) and whitefish (**b**) with abdominal cavity partly visible to expose the swim bladder, which is tapered in the stickleback but rounded in the whitefish. Drawings by Leonie Kneipp, LKdraw.

Fish used for the experiments were 90 wild caught juvenile and adult sticklebacks measuring 5.1 cm ± 0.7 cm TL (mean ± SD), the most common size for the season, and 90 hatchery-reared juvenile offspring of wild whitefish (7.0 cm ± 0.6 cm TL). Sticklebacks were obtained from a trawl net fishery in Lake Constance shortly before the experiment began and kept in 240 L flow-through basins supplied by lake water until the start of the experiment. Water temperature and oxygen concentration were measured twice daily and sticklebacks were fed once a day with chironomid larvae. The whitefish were hatchery-reared offspring of wild spawners obtained from the Fish Hatchery of the Stocking Commission in Langenargen, Germany. The fish were kept in the hatchery and fed with commercially available dry food until needed for the experiment. The two species differ in body covering and the shape of the swim bladder: sticklebacks have bony plates and a swim bladder with tapered ends while whitefish are fully scaled with a rounded swim bladder (Fig. 2). In both species the swim bladder length to body length ratio is similar, around 22% (± 2.5% SD) for stickleback and 24% (± 3% SD) for whitefish.

Lake water temperature remained constant at 16 °C (± 0.03) and oxygen saturation was 100% (± 0.02) throughout the experiments. By the end of each experiment, all fish were carefully captured from the mesocosm, anaesthetised with clove oil and measured to the nearest mm of body length. After recovering in an aerated tank supplied with lake water, they were then restocked into the lake.

All experiments were conducted according to the German Animal Welfare Act (TierSchG) and approved by the ethics committee of the Referat Tierschutz of Regierungspräsidium Tübingen (LAZ 2-18, AZ 35/9185.81-4).

### Software

Data was recorded using Simrad’s standard operating software EK80 (Ver. 1.10.1) configured to save .RAW files. Post-processing was carried out with Sonar5-Pro (S5) suite (Ver. 605.0) from Balk and Lindem^51^ in combination with python (Ver. 3.7.3). The numerical feature descriptor in S5 and the utilized python script were custom developed for this study and are either available in the latest version of S5 or can be requested by the corresponding author, respectively. Species classification was performed in R (Ver. 3.4.0^52^) using the randomForest package (Ver. 4.6-14^53^), and differences in species classification methods were compared using a t-test and a Fisher’s exact test for count data in R.

### Hydroacoustic data collection

Hydroacoustic data were collected using an EK80 wide band echosounder equipped with an ES-120-7C split beam transducer, emitting a nominal frequency of 120 kHz with an opening angle of 7 × 7 degrees. The transceiver was set up in FM mode with fast ramping for the envelope, which tapers the first and last two wavelengths of the emitted signal over a duration 0.0434 μs, 100 W emissions (electric power), a pulse duration set to 0.512 ms, and a ping rate of ~ 9.5 pings per second. The bandwidth of the chirp was set to 80 kHz, ranging from 90 to 170 kHz. This frequency range was chosen because it is centered around 120 kHz, which is commonly used in fish hydroacoustics^54,55^. A short pulse duration was used to allow sampling of clean echoes from single targets under the high density mesocosm conditions. Prior to data collection the system was calibrated using a 23 mm copper sphere and the calibration option in Simrad’s standard operating software EK80 (Ver. 1.10.1). Data collection started as soon as fish were introduced to the net cage and stopped just before they were taken out.

At midday, 30 live sticklebacks were introduced carefully into the net cage. After three hours the sticklebacks were removed from the mesocosm, and 30 live whitefish were introduced for another three hours, after which data collection was stopped and all fish were removed from the mesocosm. The experiment was repeated on the subsequent 2 days.

Individual, fully sonified, fish targets were identified in the echograms and ten frequency responses were extracted from each target to accommodate possible intra fish variation. Each frequency response was linearized and normalized, which produced a frequency response curve (FRC) consisting of 656 amplitude samples along the frequency-axis from 90 to 170 kHz (referred to as TS_u_(f) in^45^). The frequency spectra were normalized to be able to compare the relative spectra without influence of echo intensity. For species with clear difference in echo size, classification based on TS is simple. For sticklebacks and whitefish, however, there are overlapping size classes with similar TS distributions. Moreover, with normalized spectra we could extract signals from the targets directly without including complicated single echo detections and off-axis compensation. This full FRC was used for classification, and was also used to develop a subset of features thought to support classification, referred to as the numerical feature descriptor (NFD). More details on hydroacoustic data processing and development of the NFD are given in the “Supplementary Information”.

Frequency responses were extracted from 101 free swimming sticklebacks and 86 free swimming whitefish. Of these, 1400 frequency responses resulting from 10 observations of 74 sticklebacks and 66 whitefish targets from experimental days one and three were used to train the model. The 470 frequency responses from the remaining 27 sticklebacks and 20 whitefish targets collected on experimental day two were used as the testing dataset.

Orientation or target aspect angles has been shown to greatly alter narrowband hydroacoustic responses of individual targets and is likely to affect wideband frequency responses. Seen from the dorsal aspect a targets echo will be strong and short. Tilting the target will cause the echo to be weaker and longer with smoother flanks relative to the echo from the dorsal aspect. According to Fourier theory, this change will influence the frequency response as energy from the high end of the frequency spectra is shifted towards the lower end. S5 was used to track fish and measure aspect angles, using the average aspect method (see “Supplemental Information” for further details). The absolute orientation angle was measured relative to the surface (i.e., horizontal was 0° and vertical was 90°). Orientation or aspect angle was then classified as having a low (0–20), medium, (20–40) or high (> 40) angle. The effect of orientation on hydroacoustic responses was visually assessed and its effect on classification accuracy was inspected as described in the section below.

### Classification of fish species

Species classification was done using random forest, which is an ensemble of classification trees developed through feature subsampling and bootstrapping across training data^56^. The random forest classifier was used because it has shown good performance for diverse classification and regression problems in ecology^57,58^ and no dimensionality reduction was necessary for using the FRC. In addition, random forest is able to estimate feature importance among a set of highly correlated features, such as those from the FRC, by subsampling *m* features at each split, provided that *m* is not too large and a sufficient number of classification trees is fit so that all features are subsampled multiple times^56,57^. The default value for m is the square root of the total number of predictors rounded to the nearest integer, which was used since preliminary analyses showed that it provided optimum classification accuracy. Since a sufficient number of classification trees is needed for accurate estimate of variable importance and forests do not become overfit with too many^56^, 5000 trees were trained in each forest instead of the default 500. Default values were used for all other tuning parameters.

Random forests were fit using both FRCs and NFDs to see which approach provided better accuracy. Since the ten observations were taken from each fish target are correlated, the simple random splitting procedure of random forest would likely have overestimated classification accuracy for new fish. To accurately estimate the accuracy of classifying observations from new fish during model training, cross validation was performed using all ten observations from 90% of fish selected at random from the training data set to fit the model, then using the remaining observations from the withheld fish to assess accuracy. The cross validation procedure was repeated 30 times using the train function of the caret package in R (version 6.0–81^59^). Species classification of observations from new fish was assigned based on majority voting across all trees in the forest. The final random forest was then fit using all training data and accuracy was assessed for the testing data. To better understand the contribution of specific NFD features or FRC frequencies for species differentiation, feature importance was measured as the average decrease in node impurity as measured by the Gini index across all trees in the forest^53,60^.

To determine the effect of orientation on classification accuracy, training and testing datasets were first combined to increase sample size. Classification accuracy with random forest was then calculated within each of the orientation classes using cross validation as described above. There were insufficient numbers of both species in the high orientation group to assess accuracy (see “Results”).

The intra- and inter-individual variation in frequency responses was quantified for each species to enable comparisons. The normalized FRC was first logit transformed to enable calculation of unbounded deviations. The intra-individual deviations at each frequency were calculated as the difference between the values at each of the ten responses and the individual target’s mean values. The inter-individual deviations were calculated as the difference between each individual’s mean value and the species’ mean value. The mean absolute deviation was then calculated for each species and frequency, as well as summarized across all frequencies to give a grand mean absolute deviation.

## Results

**Figure 3:**
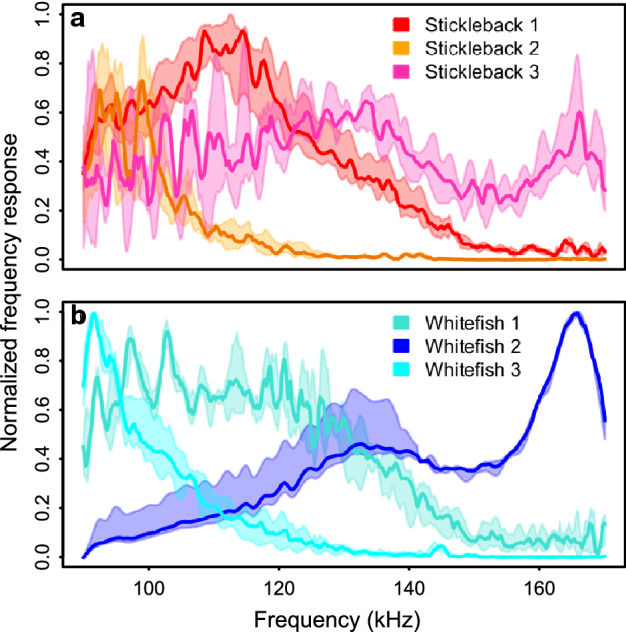
Variation in relative frequency response curves (FRC) from three individual sticklebacks and whitefish. The bold line is the median and the shaded polygon region includes the 25–75 percentiles for observations from each fish, where overlapping regions are shown by the combined colours.

**Figure 4:**
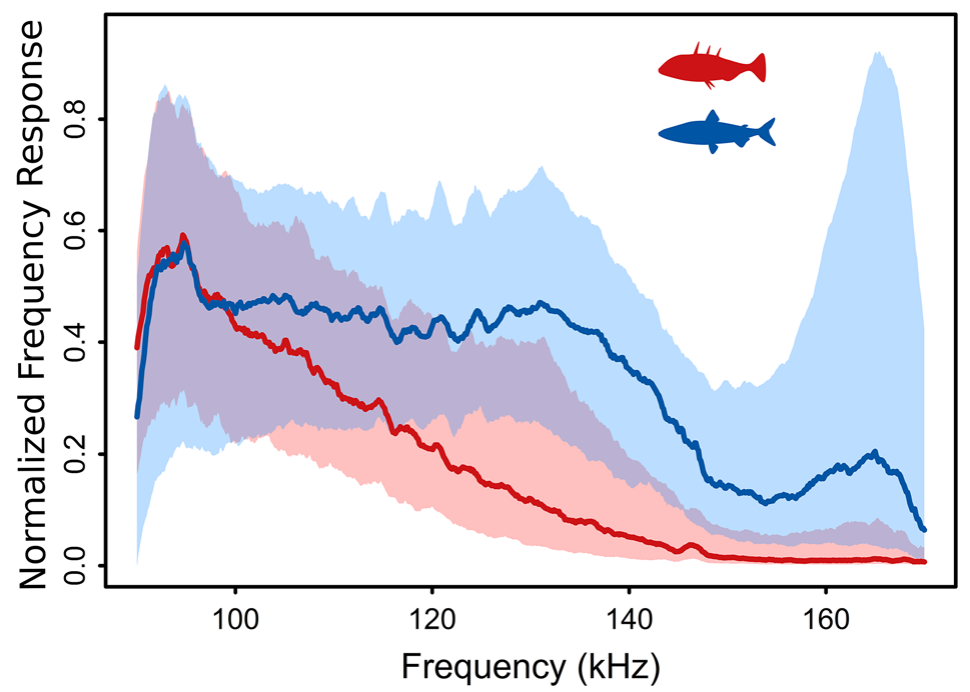
Comparison of the relative frequency response curves (FRCs) for stickleback (red) and whitefish (blue). The bold line is the median and the shaded polygon region includes the 25–75 percentile observations from all individuals of both species, and overlapping regions are shown by combined colours.

There was a high degree of variation in FRCs within a single target, among targets, and between species. Combined plots of FRCs from three randomly selected sticklebacks and whitefish also show that the frequency response can vary greatly over the ten responses taken for a single target, as the interquartile range is often quite large (Fig. 3). A similar plot comparing FRCs of both species reveals a large overlap at lower frequencies (< 120 kHz) but greater differences in the 135–170 kHz range (Fig. 4).

The intra- and inter-individual variation summarized across all frequencies were of a similar magnitude for whitefish (MAD = 0.94 and 0.99, respectively), but differed for stickleback (MAD = 0.81 and 1.33, respectively). Intra-individual variation was slightly higher at both ends of the frequency range for both species, but otherwise showed no clear trends. Inter-individual variation showed an increasing trend from 110 to 160 kHz for stickleback, and a similar trend from 120 to 160 for whitefish. From 160 to 170 kHz the trend tended to decrease for both species (Fig. S6).

**Figure 5:**
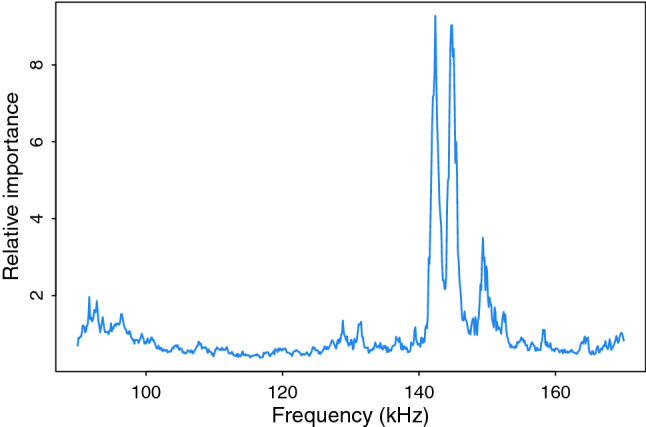
Relative importance (measured by the Gini index) of the different frequencies from 90 to 170 kHz for random forest classification of stickleback and whitefish.

Classification accuracy from the training data was slightly but significantly higher for the FRC compared to the NFD method (t-test, 1400 frequency responses, t-value = 3.03, df = 57.7, p-value = 0.0036; Table 1). Classification accuracy for the testing data set was also significantly higher for the FRC method (Fisher’s exact test, 470 frequency responses, p-value = 0.027). Frequency responses for whitefish were correctly classified more often than those for sticklebacks, using both the FRC (Fisher’s exact test, p-value < 0.001) and NFD (Fisher’s exact test, p-value < 0.001; Table 2). The positive predictive value for whitefish was 83% (166/200) using the FRC, but only 70% (140/200) using the NFD. Positive classification of sticklebacks differed only slightly between FRC and NFD (Table 2). Higher frequencies of the acoustic band showed the greatest potential for acoustic species identification using random forests, with a relatively narrow range between 140 and 155 kHz receiving most of the variable importance as measured by the Gini index (Fig. 5).

**Table 1:** Overall accuracy of the random forest models developed for classifying stickleback and whitefish using numerical feature descriptors (NFD) and the frequency response curves (FRC) estimated for training data through cross validation (CV accuracy) and for test data (Test accuracy). For CV accuracy, the mean accuracy and standard deviation was estimated through tenfold cross validation with three repeats.

Prediction approach	Train accuracy (SD)	Test accuracy (%)
NFD	73.8% (7.6)	66.6
FRC	78.1% (6.8)	73.4

**Table 2:** A confusion matrix showing species classification of targets from the testing data set using two prediction approaches, frequency response curve (FRC) and numerical feature descriptor (NFD). Each cell contains the number of known stickleback or whitefish responses (rows) that were predicted to belong to either stickleback or whitefish (columns).

	Predicted identity			
	Frequency response curve (FRC)		Numerical feature descriptor (NFD)	
True identity	Stickleback	Whitefish	Stickleback	Whitefish
Stickleback	179	91	173	97
Whitefish	34	166	60	140

Stickleback and whitefish targets had similar orientation angles and were most often low or medium, with only eight sticklebacks and six whitefish targets having high orientations (Table 3). The frequency responses of the two species within the low and medium orientation were similar to those from all fish (“Supporting information”, Figs. S3–S5). Frequency responses appeared somewhat different in the high orientation group, but low sample sizes of stickleback and whitefish make these comparisons tenuous. Cross validation based estimates of classification accuracy within the low (79.0 ± 7.9%) and medium (76.7 ± 10.3) orientation group were similar and within the expected accuracy range estimated using the training dataset across all orientations (78.1 ± 6.8%). There were too few targets in the high orientation groups to provide an accurate assessment of classification accuracy.

**Table 3:** Number of stickleback and whitefish in each of the orientation different orientation classes, measured as absolute degrees relative to the water surface.

Orientation class	Stickleback	Whitefish
Low (< ± 20°)	67	48
Medium (≥ 20° & < 40°)	26	32
High (≥ 40°)	8	6

## Discussion

This study shows the potential for wideband hydroacoustics to enable species differentiation of similar-sized, swim-bladdered individuals. A recent study^45^ described wideband acoustic responses for schools of several species encountered during trawls, and concluded that differentiation of multiple species with swim bladders would be challenging due to similarities in acoustic responses. Acoustic responses of whitefish and stickleback individuals were also similar in this study, but classification was achieved using random forests with reasonably high accuracy. Most previous efforts to classify species using hydroacoustic monitoring have focused on differentiating schooling species with morphometric and bathymetric descriptors of school behaviour (reviewed in^14,^^45^). For example, single species schools of anchovy, sardine, and round herring were differentiated using morphometric, energetic, and bathymetric traits derived from echograms with discriminant function analysis^61^ and artificial neural networks^20^. The ability to identify the species identity of individual targets with wideband hydroacoustics could expand these capabilities to the much larger number of species that do not display strong schooling behaviours. In addition, single-species aggregations in mesocosms were successfully differentiated by hydroacoustic data collected using custom-designed wideband echosounders in previous studies^29,62^. This would suggest that schooling or shoaling species may also be differentiated using widely available commercial wideband echosounders, but this remains to be tested.

The model developed in this study, once verified with field data, will expand monitoring capabilities so that both populations of stickleback and whitefish can be better monitored in Lake Constance. The likely invasion timing and potential abundance of sticklebacks was estimated through hydroacoustics by Eckmann and Engesser^32^ by assuming that the abundance of other small pelagic fishes did not increase. Classifying small fish targets with the tool developed in this study will provide more accurate estimates of stickleback and juvenile whitefish abundance to help inform species spatial overlap, interspecific competition, abiotic preferences (e.g. temperature), and foraging. Information pertaining to the seasonal distribution of stickleback could help to better define spawning migration into near-shore areas^63^, and this information might be utilized to alter the timing of larval whitefish stocking in order to limit predation^49,64^. Abundance estimates for juvenile whitefish provided by hydroacoustics may also help improve estimates of natural recruitment and stocking success.

The mesocosm experiment was carefully designed to collect data of free swimming individuals within a lake environment to enable its transferability to the lake environment. Previous studies have shown that orientation of targets can affect acoustic responses and TS measurements, as well as population estimates^65^. Orientation of targets could also alter the FRC and classification accuracy if unaccounted for. In this study, individuals of both species were mostly found with orientations below 40° relative to the water surface, with a few individual targets at higher angles. Classification accuracy of individuals that exhibited low and medium orientations was similar to the expected bounds of accuracy estimated for all orientations. The results suggest that classification accuracy does not differ greatly with orientation, but additional data collected on individuals with high orientations is needed to determine the limits of this conclusion. It is expected that juvenile whitefish and sticklebacks have similar orientations in the lake environment to those free-swimming in the mesocosm, but this will also need to be considered in field applications.

Orientations of targets was estimated using tracked motions, which requires that fish are actively swimming to give precise measurements. Since only targets entering and leaving the narrow acoustic beam were used in this study, all included fish had to be actively swimming. Some targets, however, moved too little between individual observations to give reliable orientation estimates. This was most profound for stickleback. Since classification accuracy was similar for almost all fish regardless of orientation, it is not expected that this uncertainty would greatly affect our general conclusions. Future studies investigating the effect of orientation on wideband acoustic responses would benefit from using paired cameras or other methods to more accurately measure orientation.

In addition to potential variation from orientation, there was substantial intratarget variation among hydroacoustic responses within an individual fish target that stemmed from extracting data from different parts of the fish echo. This study has not attempted to determine the underlying reasons, but these could presumably be due to how much swim bladder area is sampled, the presence of plating on some parts of the body in stickleback, or other morphological differences along a single fish. Accuracy for whitefish was higher than for sticklebacks, which could reflect greater morphometric variation in the latter which is indeed shown for Lake Constance sticklebacks^66^. This study used wild caught sticklebacks with different plate arrangements that are largely representative of those in the lake. For these reasons it is expected that the model will have similar accuracy across fish of different orientations, sizes and stickleback plating patterns in field tests.

Previous studies have used other machine learning and statistical classification approaches, including artificial neural networks (e.g.^20,21,28^), support vector machines (e.g.^21^), and discriminant analysis (e.g.^62^) for hydroacoustic classification. Random forests were used in this study because of its superior performance in tackling a diverse range of classification and regression problems in ecology^57,58^. In addition, random forests could be trained using the full FRC without the pre-processing of data or the dimensionality reduction required for these other methods. This ability was important as the FRC were shown to generate greater classification accuracy than the NFD in this study. Other features or a subsample of frequencies may provide similar classification accuracy^37^, but these summaries could not include more information than the FRC and the effort to select such features seems unnecessary when used with random forests or related approaches.

While wideband hydroacoustic classification may have great potential for monitoring multiple species, there are difficulties in collecting the data needed to develop classifiers for individual targets. This is especially true in a mixed species experiment or under field conditions, since the species identify of individual targets needs to be known. A mixed species experiment was also attempted in the current study in which sticklebacks and whitefish were held together in the mesocosm overnight, and video cameras were used to record individual fish location and identity. However, the data from this experiment could not be used to further test the classifier due to poor visibility below 3 m depth, which prevented species identification. One potential solution for future mesocosm or field studies is to use unique fluorescent marks so that species can be identified using video, and possibly also using hydroacoustic tracking to ensure that the correct individual is identified in the echogram. Another solution with sufficiently large fish would be to tag one or more species, identify tagged individuals using acoustic data, and then filter out the acoustic response from the tag. In the current study a relatively small amount of the mesocosm was covered by the acoustic cone to avoid interference from mesocosm sidewalls, which reduced the likelihood of recording individual targets and limited data. Future mesocosm experiments may benefit from increasing cone coverage to maximize data collection, provided that interference is avoided.

It is expected that further studies will determine the extent to which wideband hydroacoustics can be used to identify other fish species. Such studies could enable species classification and greatly improve monitoring of fish community dynamics through noninvasive sampling. When combined with the real-time data processing capabilities of software, such as Sonar5-Pro, classification algorithms may also facilitate species-specific fishing in order to reduce bycatch and maximize the sustainability of fisheries^14,61^.

## Supplementary Information

Supplementary Information.

## Data Availability

The datasets generated and analyzed during the current study are available from the corresponding author on request.
