# Supplementary material for: Differentiation of two swim bladdered fish species using next generation wideband hydroacoustics
Source: Sci Rep. 2021 May 18;11:10520. doi: 10.1038/s41598-021-89941-7 (PMC8131709; doi:10.1038/s41598-021-89941-7)
Supplement: Supplementary file 1 — Supplementary Information. [file 41598_2021_89941_MOESM1_ESM.pdf]

Differentiation of two swim bladdered fish species using next generation wideband hydroacoustics

Sarah M. Gugele[a, c, *], Marcus Widmer[b], Jan Baer[a], J. Tyrell DeWeber[a], Helge Balk[b], Alexander Brinker[a, c]

[a] Fisheries Research Station Baden-Württemberg (LAZBW), Argenweg 50/1, 88085 Langenargen,Germany

[b] University of Oslo, Department of Physics, PO. Box. 1048. Blindern, NO-0317 Oslo, Norway

[c] University of Constance, Institute for Limnology, Mainaustraße 252, 78464 Konstanz, Germany

Corresponding author: Jan Baer, Fisheries Research Station Baden-Württemberg, Argenweg 50/1, 88085 Langenargen, Germany, jan.baer@lazbw.bwl.de

Running headline: Fish species differentiation via hydroacoustics

Supporting information

Hydroacoustic data processing

Sonar5-Pro allows the operator to click on a selected target in an echogram with the mouse and extract hydroacoustic data. After a thorough investigation of hydroacoustic data from sticklebacks and whitefish Sonar5-Pro was set to extract acoustic data covering 150 % of the emitted pulse length, vertically symmetrical around the clicks to ensure that frequency responses included all energy from the selected target. The 150% buffer around the target may have included some signal from surrounding water, but this would have little effect on the signal since echo from water is two orders of magnitude lower than fish (Fig. S1).

a)

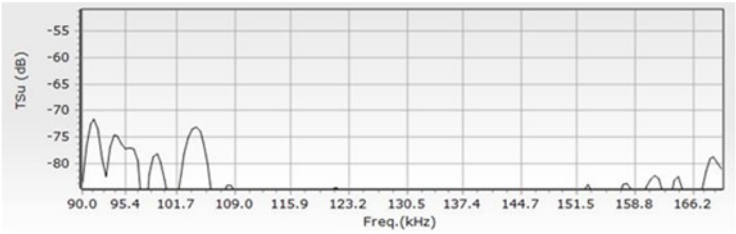

b)

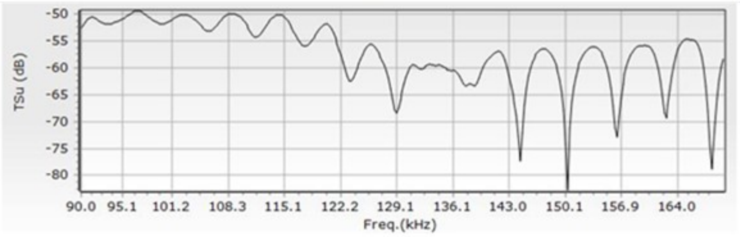

c)

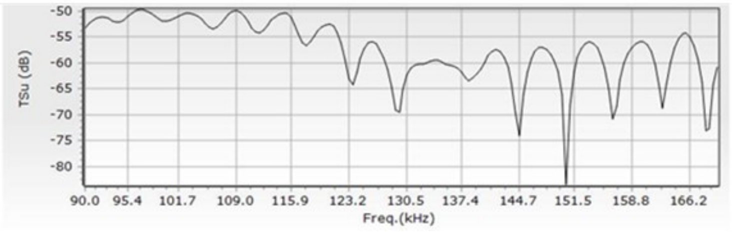

Supplementary Figure S1: FRC of a) free water (buffer 100 %), b) whitefish (buffer 150 %), c) whitefish (buffer 100 %).

A mouse click on a target extracts a signal piece consisting of 96 samples from the underlying complex acoustic raw data. Processing this signal with a Fast Fourier Transform (FFT) produces

the frequency response. Adding zeros (traditional zero padding in signal processing) was applied to meet the FFT's requirement for the number of samples to be 2^N. The FFT algorithm can for example handle sample sets of 2^7=128, 2^8=256, or 2^9=512 samples. We applied 2^9 because this produces TSu(f) functions with the same smoothness as presented by Simrad's EK80 software. Due to the EK80 sampling regime aliasing occurs and the output from the FFT must be split and reorganized to produce the frequency spectra. Due to EK80's sampling regime, aliasing occur and the output from the FFT must be split and reorganized to produce the frequency spectra. This reorganized spectral density is too weak in the beginning and the end due to the algorithm modifying the intensity of the frequency response in accordance with the intensity of the emitted pulse (called ramping in Simrads terminology). This was compensated with the inverted Fourier spectra of the modeled emitted pulse. Parameters like the absorption coefficient and transducer opening angles vary with the frequency and the algorithm therefore apply the sonar equation to each individual frequency component in the spectra. This provides the final target strength response as a function of frequency (TSu(f), given in dB). The processing details are well described by Demer et al. (2017) [S2]. The processing, as we applied it, resulted in TSu(f) functions consisting of 656 samples along the frequency-axis from 90 to 170 kHz. The subscript u in TSu(f) dB functions indicates that the TS values are uncompensated with respect to the beam pattern. We used the uncompensated target strength to avoid applying the single echo detector, which would add another layer of complexity and uncertainty to the analysis and the results. Selection of single targets had to be done manually because we needed to ensure clear water below and above each selected target to avoid contamination of the spectra by neighbour targets. Moreover, we did not use off-axis compensated responses because we were not testing if the system could obtain the true size of the target. We rather wanted to see if we could classify targets without respect to

TS and position in the beam. A surface exclusion zone was set to 2.2 m below the transducer in the software to avoid near field influence and low sampling volume [S1].

We used combined linearized and normalized frequency responses to avoid logarithmic non-linearity and the influences of fish size and beam pattern on the classification results. In the linearization process the logarithmic compression of the target strength TS is removed and the sigma $\sigma_u(f)$ [S3] is estimated instead:

Equation (1): $\sigma_u(f) = e^{\frac{TSu(f)}{10}}$

To avoid target size influencing on the classification we also normalized the $\sigma_u(f)$ to span from zero to one:

Equation (2): $\sigma_u(f)_{norm.} = \frac{\sigma_u(f) - \sigma_u(f)_{Min}}{\sigma_u(f)_{Max} - \sigma_u(f)_{Min}}$

The linearized and normalized $\sigma_u(f)_{norm.}$, referred to as the frequency response curve (FRC), consisted of 656 amplitude samples along the frequency-axis from 90 to 170 kHz. This full FRC was used for classification, and was also summarized to develop a numerical feature descriptor (NFD).

Qualitative studies of FRC from sticklebacks, whitefish, bubbles, bottom of the marina, and metal targets showed high variability in hydroacoustic responses. Some curves showed regular sinusoidal oscillating patterns with variable amplitudes, while others had more irregular patterns with a few profound peaks and nulls. In addition, curves varied in the number of peaks or dips and the existence of trends across the frequency range. Based on these observations, the NFD set of numerical features was chosen to help characterize variability among objects and potentially enable species classification. The FRC functions were input to the algorithm that detected and calculated the NFD. Table 1 and Figure 2 show the definitions of the NFD. The amplitudes of the five lowest harmonic frequencies were produced via processing the FRC with a FFT. A peak detector identified maxima in the FRC,

linear regression was applied to estimate trends in the data, and standard statistics were calculated (Table S1). The peak detector first sliced the FRC in 10 equal layers and then counted peaks in each layer, starting at the highest layer. Layers are separated by threshold lines starting at 0.9 and ending at 0.1. Figure S2 show an example on how the algorithm will work for a given FRC. We see one peak in the top layer and another peak in the second top layer. Only the first two layer lines are indicated. The process is repeated until 5 peaks has been identified or until all layers has been searched. The numerical feature descriptor is a custom developed script for this study and is available in the latest version of S5.

Supplementary Table S1: Formulas to extract the individual features of the numerical feature descriptor (NFD) vector describing the frequency response curves.

**Feature**	**Method**	**Comment**
Oscillation 1...5	Extracted by FFT	Amplitudes of the 5 lowest harmonic frequencies.
Mean	$mean = \frac{1}{N}\sum_{i=1}^{N} y_i$	
Variance	$Var = \frac{1}{N-1}\sum_{i=1}^{N}(y_i - \bar{y})^2$	
Simplified skewness	$s = \frac{1}{N-1}\sum_{i=1}^{N}(y_i - \bar{y})^3$	Similar to skewness, but with simplified normalization.

Simplified kurtosis	$$k = \frac{1}{N-1} \sum_{i=1}^{N} (y_i - \bar{y})^4$$	Similar to kurtosis, but with simplified normalization.
Nr. of peaks	Peak detector	
Trend_overall	Inclination y=ax+b	Linear regression based on all samples in the FRC.
Trend_BeforeMax	Inclination	Linear regression of the sample in the FRC from the start to the max. peak.
Trend_AfterMax	Inclination	Linear regression of the sample in the FRC extracted from the max. peak to the end of the FRC.
Trend_Interpeak	Inclination	Linear regression of the samples in the FRC extracted between the two highest peaks detected.
Peak 1..5	Frequency and amplitude	Peak detector obtaining the 5

		highest peaks and their locations.

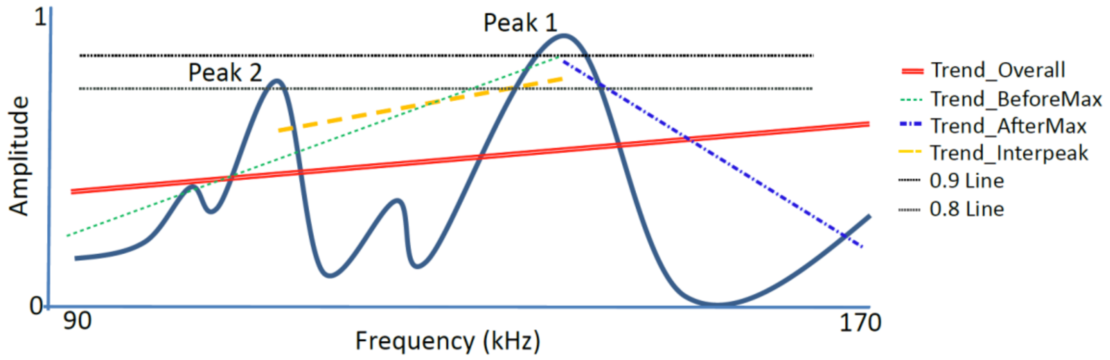

Supplementary Figure S2: Illustration of trend lines and peaks in the numerical feature descriptor extracted from a frequency response curve. The peak detector is illustrated here to highlight the two highest peaks, and further peaks would be identified as described in the text.

Each individual fish target identified in the echograms was clicked 10 times at different positions, which extracted 10 FRCs and NFDs per individual in order to accommodate intra target variation. FRC and NFD were extracted and stored as a text file via a custom developed python script that can be requested by the corresponding author.

Orientation and Frequency Responses

Built in tools in S5 were used to track each target and measure the aspect (i.e., orientation angle relative to the water surface). S5 can be set up to either find the aspect by averaging or by linear regression. We applied the average aspect method. The split beam system provides 3D positions for each individual observation within a track. Arcus tangent to the vertical and horizontal motion between neighbor observations in a track gives a series of

aspect angles. The averaging of these are output as the final aspect for each track. If we assume a track containing N observations, S5 find each ith aspect angle in the following way:

$$\Delta x_i = x_{i+1} - x_i \text{ ,}$$

$$\Delta y_i = y_{i+1} - y_i,$$

$$\Delta z_i = z_{i+1} - z_i$$

$$angle_i = \arctan\left(\frac{\Delta z_i}{\left(\Delta x_i^2 + \Delta y_i^2\right)^{0.5}}\right)$$

Finally S5 find the average aspect from all the individual aspect angles.

$$Aspect\ angle = \frac{1}{N}\sum_{i=1}^{N-1} angle_i$$

As described further in the manuscript, the orientation of each fish relative to the water surface was measured and classified as low (< ±20°), medium (≥ ±20° - < ± 40°) and high (≥ ± 40°). Figures were made to compare the FRC of each species within these orientation classes to help visually determine the effect of orientation on acoustic responses (Fig. S3-S5). The frequency responses of the low and medium classes appear very similar to those produced for all fish targets without respect to orientation (Figure 4 in the manuscript). The frequency responses in the high orientation classes appear slightly different, but this could simply be due to the very small sample sizes of 6 whitefish and 8 stickleback targets in this class.

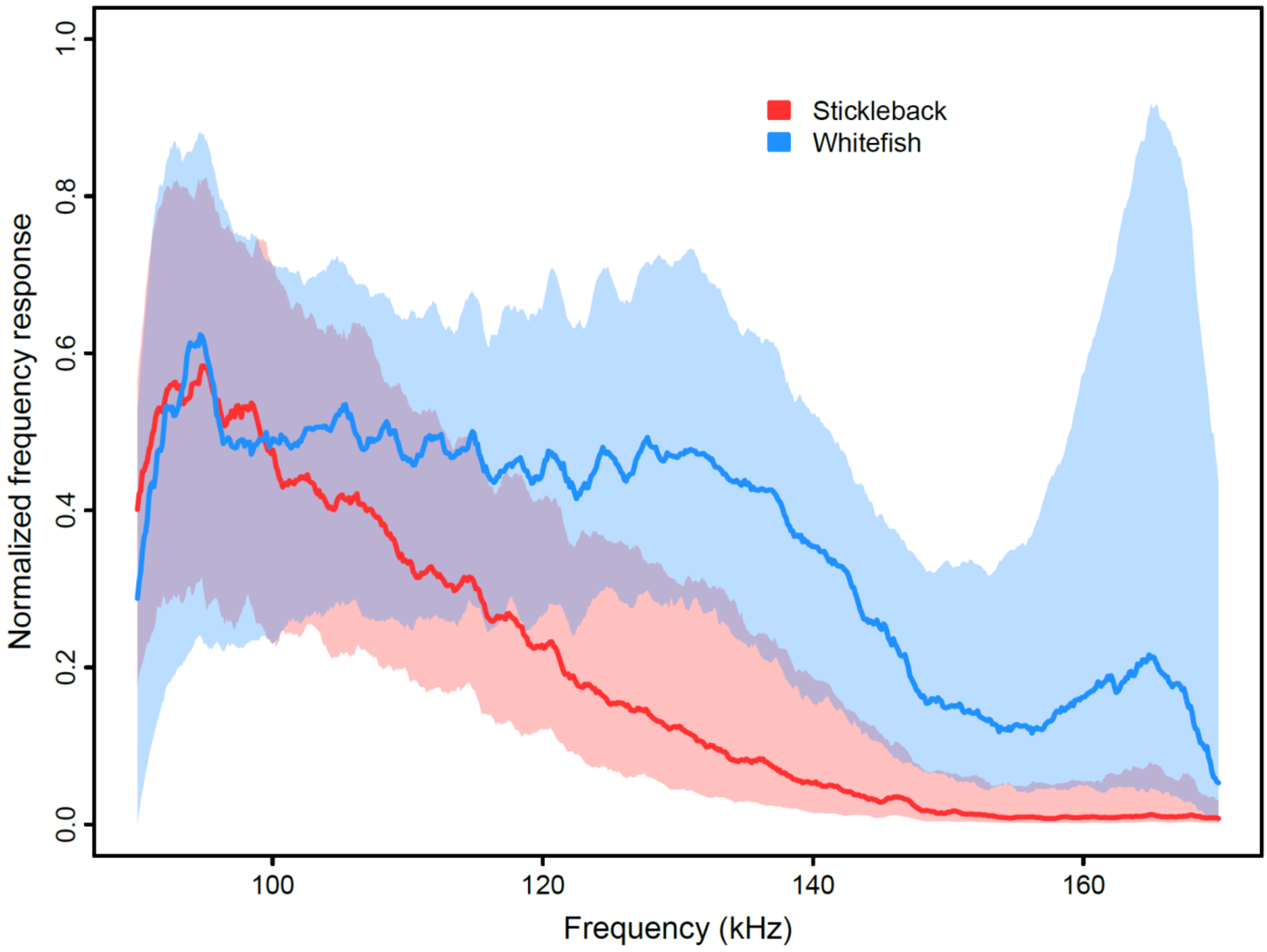

Supplementary Figure S3: Comparison of FRC for sticklebacks and whitefish with low orientations (< ±20°). The bold line is the median and the shaded polygon region includes the 25-75 percentile observations from all individuals of both species, and overlapping regions are shown by combined colours.

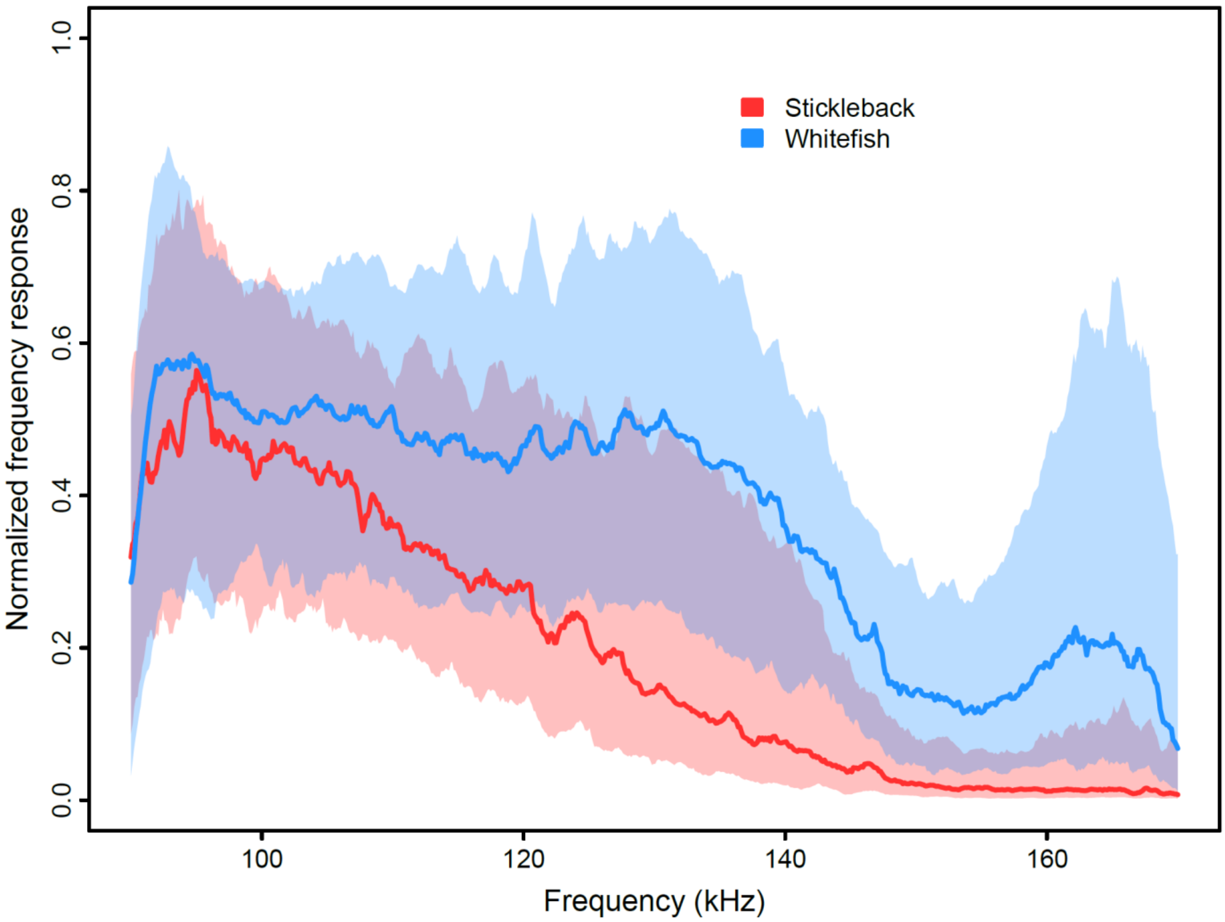

Supplementary Figure S4: Comparison of FRC for sticklebacks and whitefish with medium orientations (≥ ±20° and <40°). The bold line is the median and the shaded polygon region includes the 25-75 percentile observations from all individuals of both species, and overlapping regions are shown by combined colours.

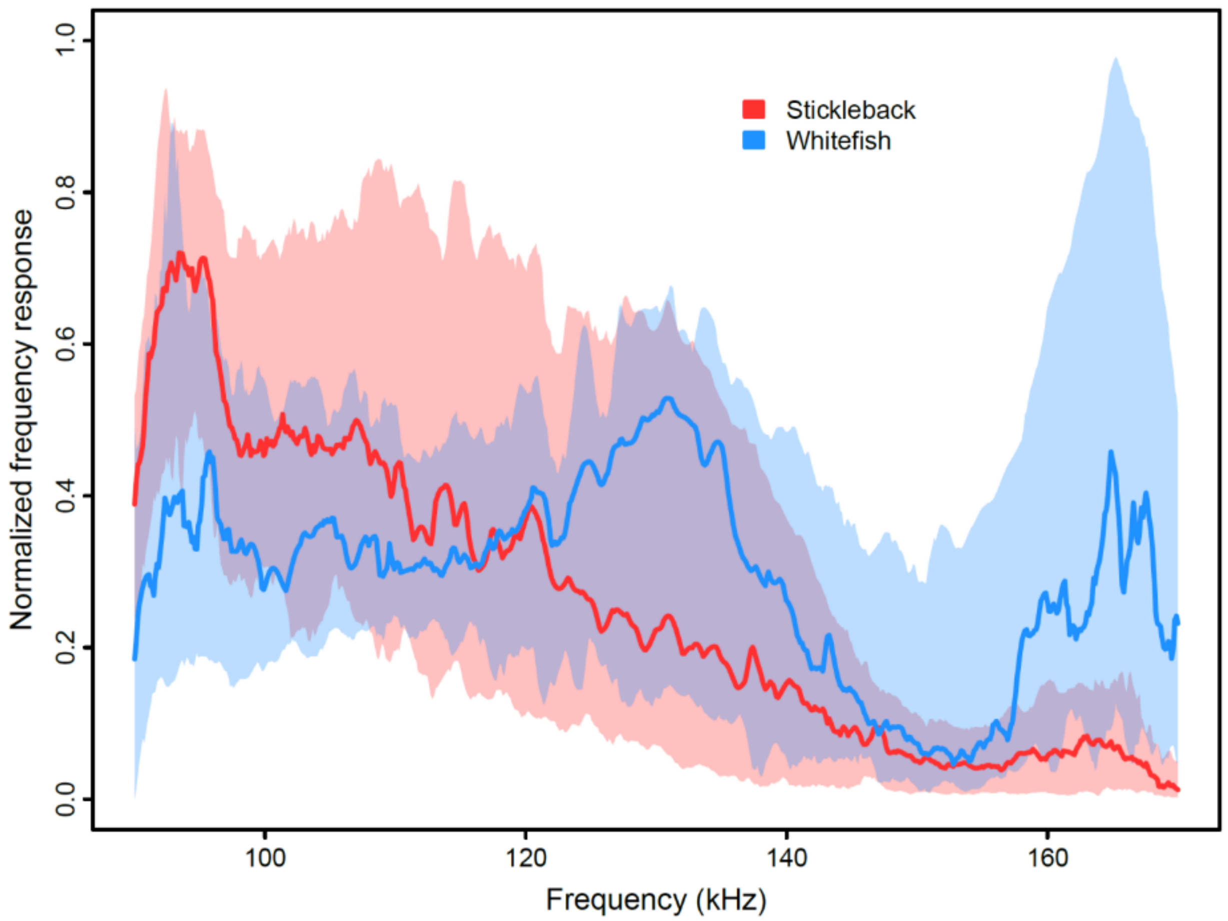

Supplementary Figure S5: Comparison of FRC for sticklebacks and whitefish with high orientations (≥ ±40°). The bold line is the median and the shaded polygon region includes the 25-75 percentile observations from all individuals of both species, and overlapping regions are shown by combined colours.

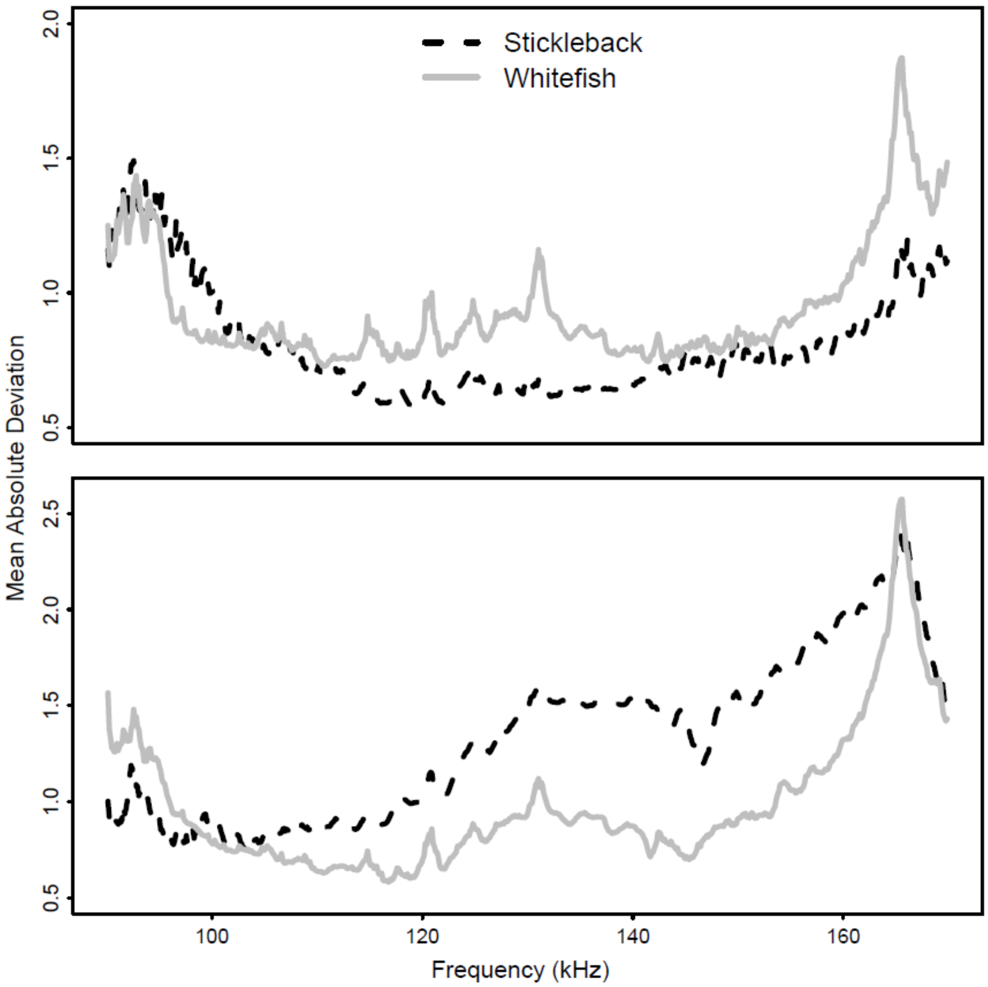

Supplementary Figure S6. The intra-individual (top) and inter-individual (bottom) variation in hydroacoustic responses measured for each species measured as the mean absolute deviation.

References

S1. Urick, R. J. *Principles of underwater sound, 3rd ed.* (McGraw-Hill Inc. USA, 1983).

S2. Demer, D. A. et al. 2016 USA–Norway EK80 Workshop Report: Evaluation of a wideband echosounder for fisheries and marine ecosystem science. *ICES Coop. Res.Rep. 336*. https://doi.org/10.17895/ices.pub.2318 (2017).

S3. MacLennan, D. N., Fernandes, P. G., Dalen, J. A consistent approach to definitions and symbols in fisheries acoustics. *ICES J. of Mar. Sci*. **59,** 365 - 369 (2002).
